# Case Report: Genomic insights into prostate adenocarcinoma transdifferentiation to carcinosarcoma due to lineage plasticity

**DOI:** 10.3389/fonc.2025.1576048

**Published:** 2025-09-03

**Authors:** Tomohiro Fukui, Arinobu Fukunaga, Yuki Teramoto, Maki Fujiwara, Kensuke Hikami, Takuro Sunada, Kei Mizuno, Yuki Kita, Takayuki Sumiyoshi, Takayuki Goto, Ryoichi Saito, Takashi Kobayashi, Shusuke Akamatsu

**Affiliations:** ^1^ Department of Urology, Kyoto University Graduate School of Medicine, Kyoto, Japan; ^2^ Department of Diagnostic Pathology, Kyoto University Graduate School of Medicine, Kyoto, Japan; ^3^ Department of Urology, Nagoya University Graduate School of Medicine, Nagoya, Japan

**Keywords:** prostate cancer, carcinosarcoma, lineage plasticity, transdifferentiation, genomic analysis, case report

## Abstract

In prostate cancer, it is recognized that adenocarcinoma can transdifferentiate into neuroendocrine prostate cancer (NEPC) owing to lineage plasticity; however, transdifferentiation into other histological types remains uncertain. We present a case of a patient who underwent surgery for adenocarcinoma, which later recurred as prostate carcinosarcoma. Genomic analysis revealed a *TMPRSS2-ERG* fusion, confirming a common clonal origin and transdifferentiation from adenocarcinoma to carcinosarcoma. Additionally, we identified a frameshift mutation in *TP53* and the loss of *PTEN* and *RB1*. Transcriptome analysis revealed enriched epithelial-mesenchymal transition and immune-related pathways, a pattern distinct from both adenocarcinoma and NEPC. To our knowledge, this is the first report that comprehensively evaluated the clonal origin of the rare prostate carcinosarcoma and characterized it using genomic and transcriptomic sequencing. It enhances our understanding of prostate cancer lineage plasticity and highlights the importance of developing novel therapies specifically targeted at prostate carcinosarcoma.

## 1 Introduction

Advanced prostate cancer is treated by targeting the androgen receptor (AR) pathway but eventually develops resistance to castration. The incidence of treatment-related neuroendocrine prostate cancer (t-NEPC), characterized by its independence from AR, is escalating due to the widespread use of next-generation androgen receptor signaling inhibitors (1, 2). Prostate adenocarcinoma is known to transdifferentiate into t-NEPC as a result of lineage plasticity; however, transdifferentiation into alternative histological types has rarely been reported (3–5). Here, we report the case of a patient with adenocarcinoma that recurred as prostate carcinosarcoma after surgery.

Prostate carcinosarcoma is an uncommon biphasic tumor comprised of a malignant epithelial (carcinomatous) component and a malignant mesenchymal (sarcomatous) component. Only approximately 100 cases have been documented in published studies (6). The diagnosis of prostate carcinosarcoma primarily relies on conventional histopathological evaluation and immunohistochemistry, as no molecular marker has yet demonstrated sufficient specificity to serve as a standalone diagnostic criterion. The prognosis is generally unfavorable, with median survival of 3 years (6).

Additionally, genomic analysis confirmed a shared clonal origin and indicated transdifferentiation through lineage plasticity. Transcriptome analysis revealed enriched epithelial-mesenchymal transition (EMT) and immune-related pathways.

## 2 Case presentation

### 2.1 Case presentation

**Figure 1:**
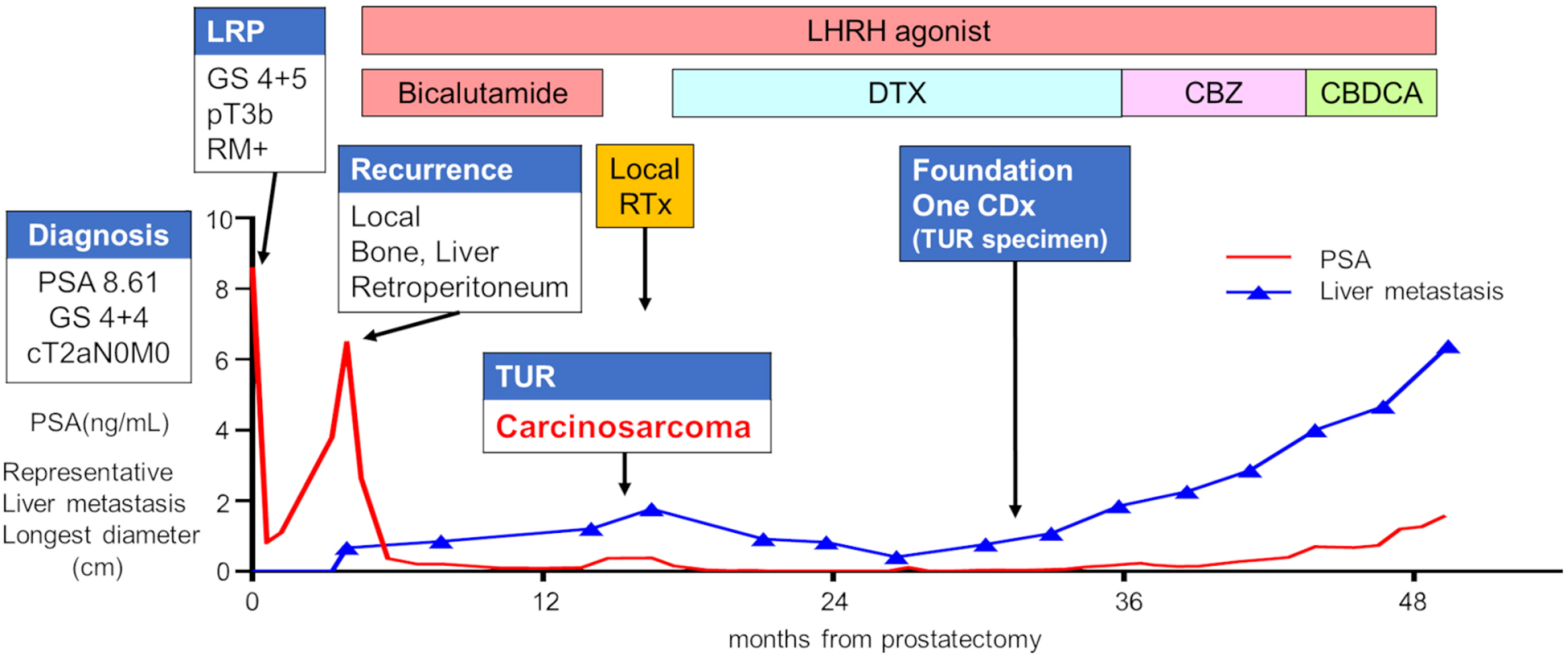
Clinical course of the patient. The red line represents the PSA value, whereas the blue line depicts the longest diameter of a representative liver metastasis tumor. CBDCA, carboplatin; CBZ, cabazitaxel; DTX, docetaxel; GS, Gleason score; LHRH, luteinizing hormone-releasing hormone; LRP, laparoscopic radical prostatectomy; PSA, prostate-specific antigen; RTx, radiation therapy; TUR, transurethral resection.

**Figure 2:**
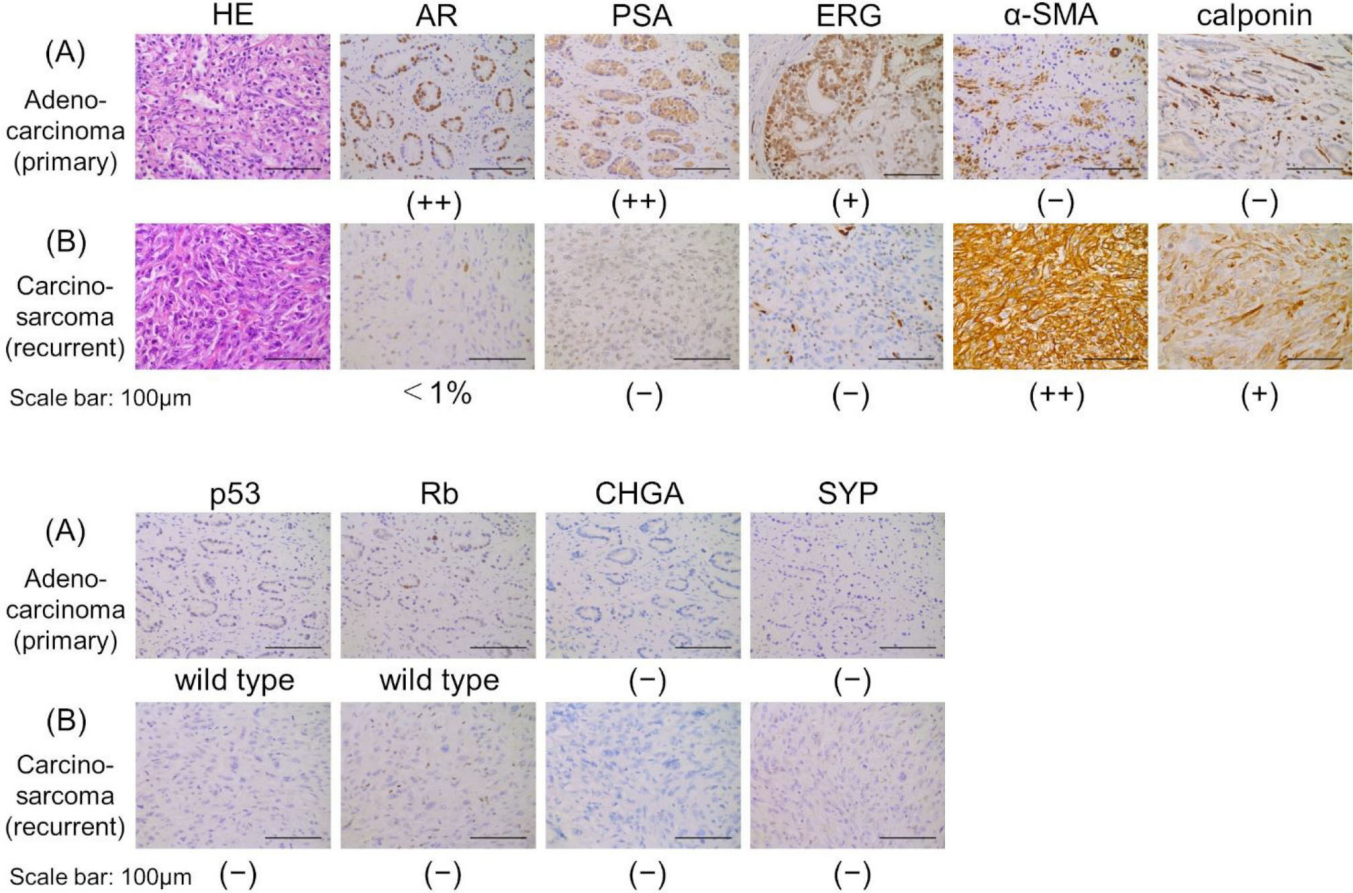
Histopathological findings of HE staining and immunohistochemistry staining. **(A)** Adenocarcinoma (primary tumor). **(B)** Carcinosarcoma (recurrent tumor). Scale bars indicate 100 µm. AR, androgen receptor; α-SMA, α-smooth muscle actin; CHGA, chromogranin A; ERG, ETS-related gene; HE, hematoxylin and eosin; PSA, prostate-specific antigen; SYP, synaptophysin.

A 64-year-old man with a prostate-specific antigen (PSA) level of 8.61 ng/mL and no relevant medical history underwent a prostate biopsy, which led to a diagnosis of adenocarcinoma (Gleason score 4 + 4), classified as cT2aN0M0 (**Figure 1**). A laparoscopic radical prostatectomy was performed, and histopathology revealed adenocarcinoma (Gleason score 4 + 5), pT3bNxMx, with positive resection margins (**Figure 2A**). However, 4 months postoperatively, the serum PSA increased from a postoperative nadir of 0.82 ng/mL to 6.48 ng/mL, and computed tomography (CT) revealed a local recurrence mass and metastasis in the liver, bones, and retroperitoneal area. The patient was initiated on androgen deprivation therapy (ADT) with leuprolide acetate and bicalutamide. Although the serum PSA level of the patient decreased, CT showed tumor enlargement.

The patient developed dysuria due to the enlargement of the local tumor, despite a low PSA level of 0.12 ng/mL. He underwent a transurethral resection (TUR) of the local recurrent tumor 11 months after starting ADT to alleviate voiding symptoms. Hematoxylin and eosin staining showed predominantly spindle-shaped cells with nuclear atypia and increased nuclear division, and immunostaining revealed only a few AR-positive cells, loss of PSA and ERG expression, and strong positivity for α-SMA and calponin. These morphological features and immunohistochemical findings led to a diagnosis of prostate carcinosarcoma (**Figure 2B**).

Immunohistochemistry staining revealed positive ERG staining in the primary tumor removed by radical prostatectomy; however, it was negative in the recurrent tumor (**Figures 2A, B**). Staining for p53 and Rb indicated a wild-type status in the primary tumor, but both were negative in the recurrent tumor (**Figures 2A, B**). Chromogranin A and synaptophysin, markers associated with NEPC, were negative in both the primary and recurrent tumor (**Figures 2A, B**).

After radiation therapy for local recurrence, the patient began docetaxel treatment. He showed a partial response after 10 months of docetaxel chemotherapy but experienced disease progression 13 months later due to new liver metastasis. After 21 courses of docetaxel, the patient received cabazitaxel, followed by carboplatin chemotherapy, but the disease continued to progress. The patient died from cancer progression 4 years and 9 months after the prostatectomy.

**Figure 3:**
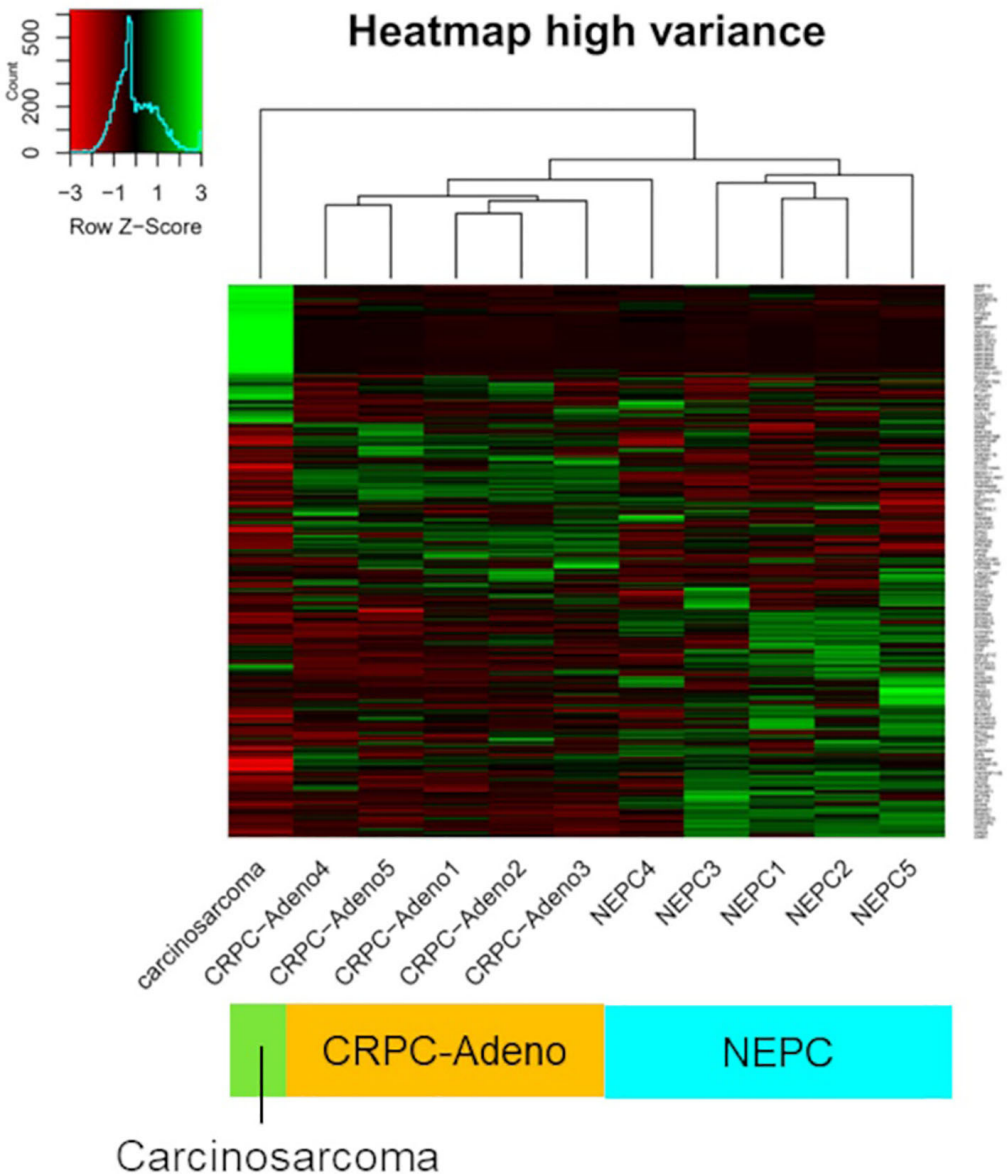
Unsupervised clustering of transcriptome data. Unsupervised clustering of transcriptome data from the carcinosarcoma (this case), CRPC-Adeno, and NEPCs using the 850 genes with the highest variance. CRPC-Adeno, castration-resistant prostate cancer-adenocarcinoma; NEPC, neuroendocrine prostate cancer.

**Figure 4:**
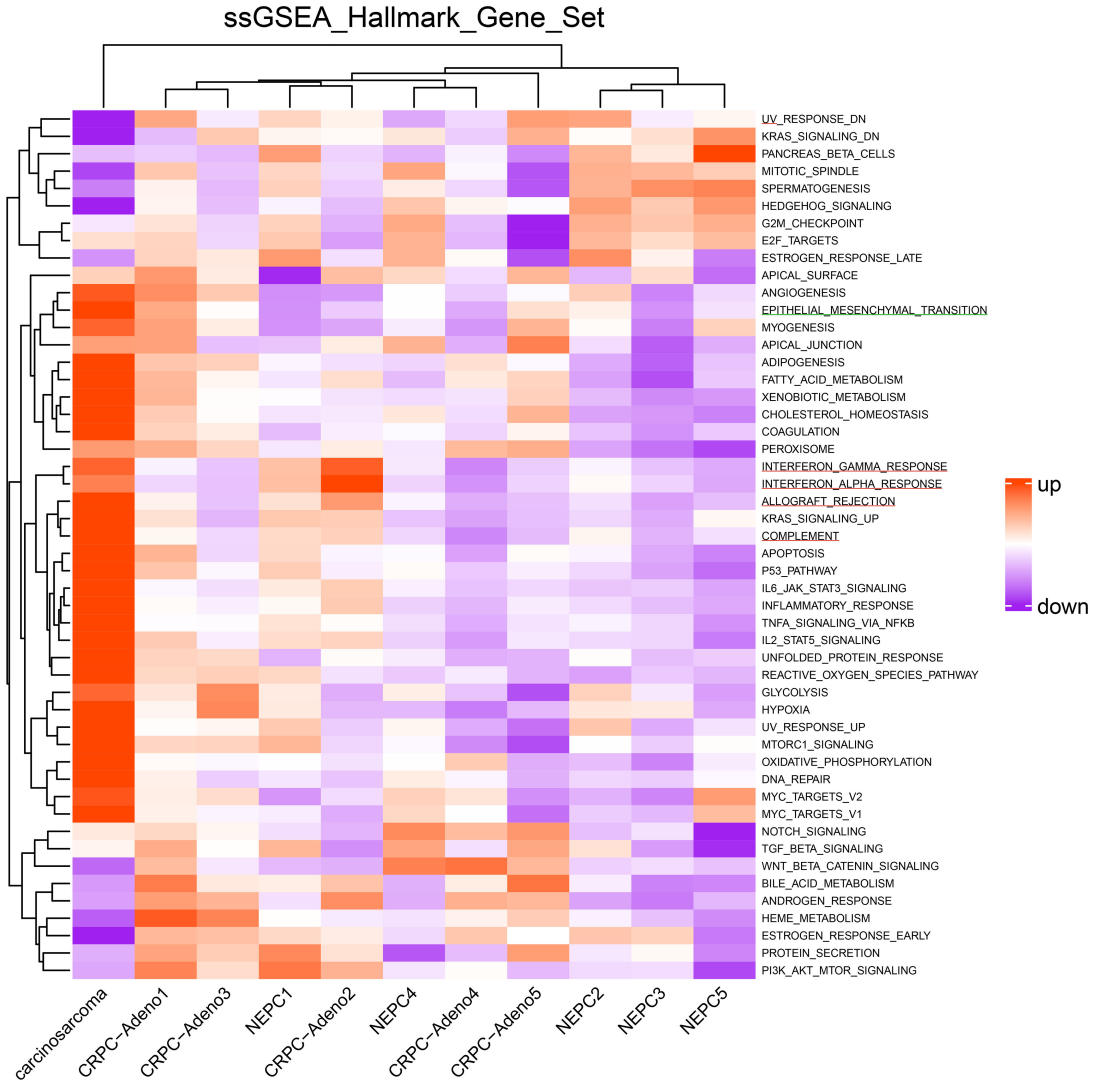
Heatmap of single-sample gene set enrichment analysis scores. Heatmap of single-sample gene set enrichment analysis scores for the hallmark gene set in the carcinosarcoma (this case), CRPC-Adeno, and NEPCs. The green line represents the EMT pathway, whereas the red line represents the immune-related pathways. CRPC-Adeno, castration-resistant prostate cancer-adenocarcinoma; NEPC, neuroendocrine prostate cancer.

**Figure 5:**
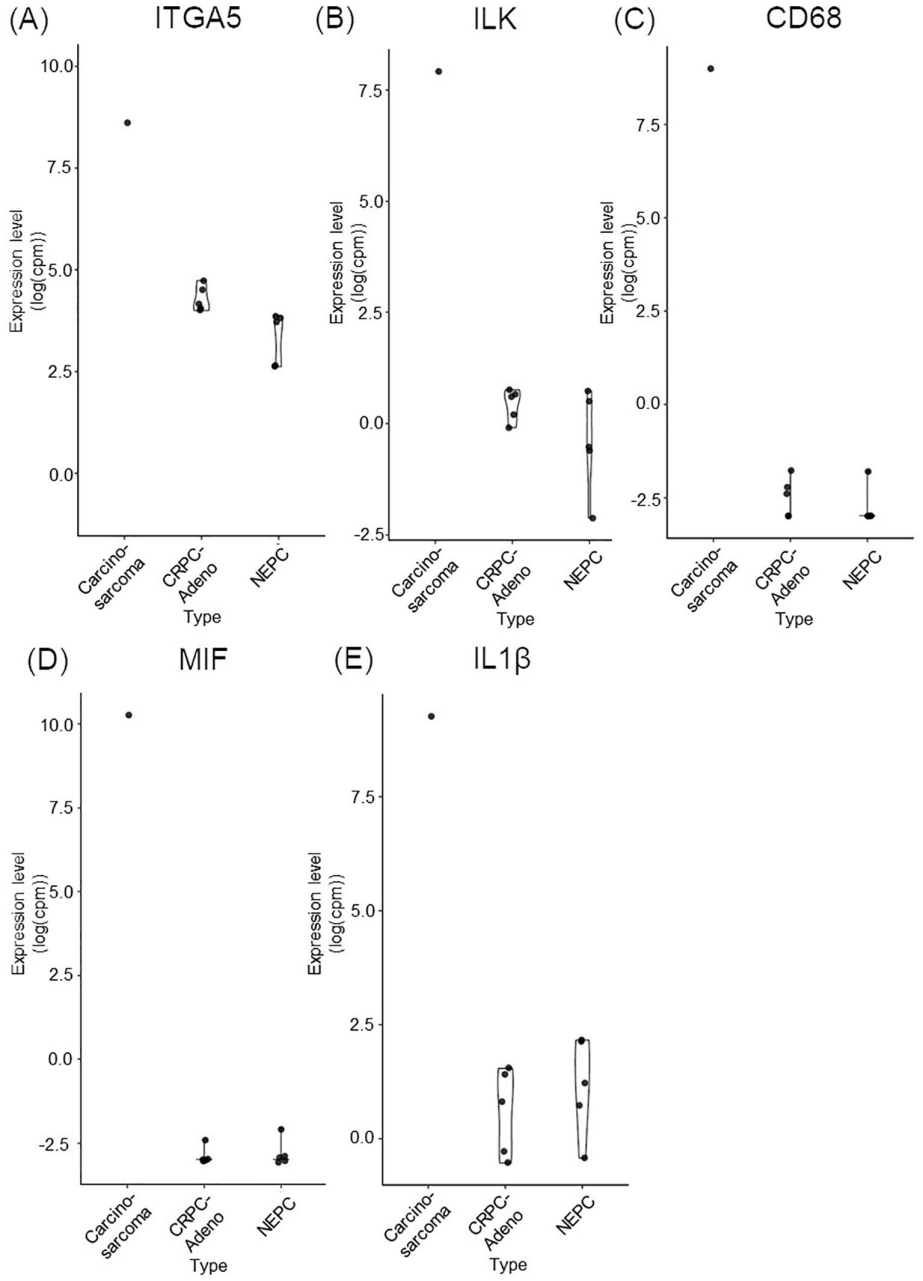
Bar plots comparing the expression of key EMT and immune-related genes in carcinosarcoma (this case), CRPC-Adeno, and NEPC. **(A)** ITGA5. **(B)** ILK. **(C)** CD68. **(D)** MIF. **(E)** IL1ß. CRPC-Adeno, castration-resistant prostate cancer-adenocarcinoma; EMT, epithelial-mesenchymal transition; IL1β, interleukin-1β; ILK, integrin-linked kinase; ITGA5, integrin subunit alpha 5; MIF, macrophage migration inhibitory factor; NEPC, neuroendocrine prostate cancer.

A FoundationOne CDx next-generation sequencing test was performed using the TUR specimen as a part of clinical care. The genomic findings are outlined in **Table 1**. The TMPRSS2–ERG gene fusion, which is specific for prostate carcinoma, was detected. Furthermore, a *TP53* frameshift mutation (T253fs*11), *RB1* exon12–17 loss, and *PTEN* loss—key genomic alterations associated with lineage plasticity leading to transdifferentiation of prostate adenocarcinoma to NEPC—were identified. Furthermore, RNA sequencing of the TUR specimens was conducted and compared with sequence data from castration-resistant prostate cancer-adenocarcinomas (CRPC-Adeno) and NEPCs reported previously (7). Hierarchical clustering analysis revealed that the present case was classified quite differently from both CRPC-Adeno and NEPC (**Figure 3**). ssGSEA showed that the EMT pathway and immune-related pathways were enriched in this case compared with CRPC-Adeno and NEPCs (**Figure 4**). Indeed, EMT-related genes (*ITGA5* and *ILK*) and immune-related genes, particularly those associated with tumor-associated macrophages (TAMs) like *CD68*, *MIF*, and *IL1β*, were remarkably highly expressed in this case (**Figure 5**) (8–10).

**Table 1:** Genomic findings of the carcinosarcoma using FoundationOne CDx.

MSI status	Stable
TMB	3 mutations/Mb
*TMPRSS2*	*TMPRSS2*-*ERG* fusion
*RICTOR*	amplification
*PTEN*	loss
*RB1*	loss exons 12-17
*TP53*	T253fs*11
*NTRK1*	G595R
*PALB2*	Q460R

MSI, microsatellite instability; TMB, tumor mutation burden.

### 2.2 Methods

Immunohistochemistry staining was performed on formalin-fixed, paraffin-embedded tissues using primary antibodies for AR (5153, Cell Signaling Technology, Danvers, MA), PSA (IS 514, Dako, Carpinteria, CA), ERG (ab92513, Abcam, Cambridge, UK), α-SMA (M0851, DAKO), calponin (M3556, DAKO), p53 (NCL-L-p53-DO7, Leica Biosystems, Buffalo Grove, IL), Rb (ab25124, Abcam), chromogranin A (LK2H10, Roche, Indianapolis, IN), and synaptophysin (A0010, DAKO). Immunohistochemical staining for AR, PSA, α-SMA, p53, Rb, and synaptophysin was performed using the avidin-biotin-peroxidase complex method, with biotinylated anti-mouse IgG antibody (dilution at 1:300; BA-2000-1.5, Vector Laboratories, Burlingame, CA, USA) and biotinylated anti-rabbit IgG antibody (dilution at 1:300; BA-1000-1.5, Vector Laboratories). Staining for ERG, calponin, and chromogranin A was automatically performed using the BenchMark ULTRA PLUS system (Roche). For antigen retrieval, citrate buffer (pH 6.0), trypsin (pH 7.5), or ULTRA Cell Conditioning Solution 1 (pH 8.5, 950-224, Roche) was used, as appropriate for each antibody. External positive control slides were not used except for Rb, as immunostaining for AR, PSA, ERG, α-SMA, calponin, p53, chromogranin A, and synaptophysin was performed using well-established protocols that have consistently produced reliable and reproducible results. Human spleen tissue was used as the external positive control for Rb immunohistochemical staining. **Table 2** summarizes the specific immunohistochemical staining conditions for each antibody.

**Table 2:** The specific immunohistochemical staining conditions for each antibody.

Antibody	Method	Dilution	Antigen retrieval solution	Incubation time	Incubation temperature
AR	ABC	1:400	Citrate buffer	overnight	4°C
PSA	ABC	1:1	Citrate buffer	overnight	4°C
ERG	automated	1:100	ULTRA CC1	32 minutes	37°C
α-SMA	ABC	1:100	None	overnight	4°C
calponin	automated	1:50	ULTRA CC1	32 minutes	37°C
p53	ABC	1:50	Citrate buffer	overnight	4°C
Rb	ABC	1:400	Citrate buffer	overnight	4°C
CHGA	automated	1:1	ULTRA CC1	32 minutes	37°C
SYP	ABC	1:50	Trypsin	overnight	4°C

ABC, avidin-biotin-peroxidase complex; AR, androgen receptor; α-SMA, α-smooth muscle actin; CHGA, chromogranin A; ERG, ETS-related gene; PSA, prostate-specific antigen4; SYP, synaptophysin; ULTRA CC1, ULTRA Cell Conditioning Solution 1.

Somatic genomics alterations were obtained using a FoundationOne CDx next-generation sequencing test as a part of the standard clinical practice (11). Total RNA was extracted from fresh frozen tissue using the RNeasy Mini Kit (Qiagen, Hilden, Germany). RNA sequencing was performed using the TruSeq Stranded mRNA Library Prep Kit (Illumina, San Diego, CA, USA) on a NovaSeq 6000 with paired-end reads of 100 bp. Raw FASTQ files were subjected to quality control using FastQC(v0.11.9), followed by adapter and quality trimming using fastp(v0.23.4). Trimmed reads were aligned to the human reference genome (GRCh38, Ensembl) using STAR (v2.7.11a). Transcript quantification was performed with RSEM (v1.3.3) using the same reference. The reference genome (FASTA) and gene annotation (GTF) files were obtained from Ensembl. Single-sample gene set enrichment analysis (ssGSEA) was performed using the calculate_sig_score_ssgsea function in the IOBR R package (v0.99.8), which is a wrapper for the gsva function from the GSVA package (v1.50.5). Hallmark gene sets were obtained from MSigDB (v2023.1). All analyses were conducted in R (v4.3.3). ssGSEA scores were Z-score normalized across samples, and heatmaps were visualized using the ComplexHeatmap package (v2.18.0).

Tissue sampling and genetic analysis were conducted with the appropriate informed consent in accordance with protocols approved by the Institutional Review Board at Kyoto University Hospital (approval number G52). The investigators obtained informed consent from the family to publish the patient’s information and images.

## 3 Discussion

In prostate cancer, lineage plasticity primarily leads to the transdifferentiation from adenocarcinoma to NEPC. The acquisition of lineage plasticity has been linked to the loss of tumor suppressor genes, including *TP53*, *RB1*, and *PTEN* (12, 13). However, the mechanisms behind lineage plasticity have not been fully elucidated, and transdifferentiation into alternative histological types has not been reported (4). Prostate carcinosarcoma is an exceptionally rare variant of prostate cancer with an unfavorable prognosis (14). Confirming the prostatic origin of prostate carcinosarcoma is challenging when it does not coexist with conventional adenocarcinoma.

In the present study, *TMPRSS2-ERG* gene fusion was identified through genomic testing, and the carcinosarcoma was confirmed to originate from prostate adenocarcinoma. This is the first report to use genomic sequence analysis to demonstrate *TMPRSS2-ERG* fusion in prostate carcinosarcoma, although a previous report identified *ERG* gene fusion in prostate carcinosarcoma using fluorescence *in situ* hybridization (15). Similar to reports on small cell carcinoma of the prostate with *ERG* rearrangements, ERG staining was positive in adenocarcinoma expressing AR but negative in carcinosarcoma lacking AR expression (16), supporting phenotypic transdifferentiation of cell lineage from adenocarcinoma.

Genomic analysis of the recurrent tumor revealed a truncating mutation in *TP53* and the loss of *PTEN* and *RB1*, representing key genomic alterations associated with lineage plasticity. Immunohistochemistry staining showed the absence of p53 and Rb in the recurrent tumor. These findings suggest that adenocarcinoma underwent transdifferentiation into carcinosarcoma through lineage plasticity.

In transcriptome analysis, carcinosarcoma was classified completely differently from NEPC, despite both undergoing common genomic changes and involving lineage plasticity. Further research is necessary to understand why NEPC and carcinosarcoma, which share genomic alterations, develop into entirely different tumors. The EMT pathway and immune-related pathway were enriched in the carcinosarcoma. Carcinosarcomas are predominantly observed in female reproductive organs, specifically the uterus and ovaries (5). In ovarian carcinosarcoma, the sarcomatous component is reported to originate from the carcinomatous components through EMT processes (17). During EMT, cancer cells exhibit plasticity regulated by epigenetic mechanisms (18). Carcinosarcomas are recognized as tumors closely associated with lineage plasticity through EMT (5). Moreover, TAMs play a crucial role in the progression of EMT in tumor cells through their interactions, potentially influencing the transition to carcinosarcoma (8).

Prostate carcinosarcoma lacks a standardized systemic treatment and typically carries a poor prognosis. In the present case, docetaxel initially suppressed tumor growth for approximately one year, but subsequent chemotherapies were ineffective. This underscores the critical need for novel treatments for prostate carcinosarcoma.

In ovarian carcinosarcoma, targeting EMT with eribulin has been reported to enhance sensitivity to immune therapy (17). In sarcomas and carcinosarcomas in general, immunotherapy is emerging as a promising approach for future treatment strategies (19–21). Furthermore, targeting TAMs, which play a role in immunosuppression during tumor development, represents another potential therapeutic avenue (21).

In conclusion, this case represents the first documented instance of prostate carcinosarcoma conclusively shown to originate from adenocarcinoma through genomic analysis. This report significantly enhances our understanding of prostate cancer lineage plasticity and the importance of developing novel therapies specifically targeted at prostate carcinosarcoma.

## Data Availability

The sequencing data for CRPC-Adeno and NEPCs are existing datasets. Publicly available datasets were analyzed for CRPC-Adeno and NEPCs. This data can be found here: dbGAP (phs001648). The sequencing data of prostate carcinosarcoma will be made available by the authors, without undue restriction.

## References

[1] Aggarwal R Huang J Alumkal JJ Zhang L Feng FY Thomas GV. Clinical and genomic characterization of treatment-emergent small-cell neuroendocrine prostate cancer: A multi-institutional prospective study. J Clin Oncol. (2018) 36:2492–503. doi: 10.1200/JCO.2017.77.6880, PMID: 29985747 PMC6366813

[2] Zhu J Liang X Wu D Chen S Yang B Mao W. Clinicopathological characteristics and survival outcomes in neuroendocrine prostate cancer: A population-based study. Medicine (Baltimore). (2021) 100:e25237. doi: 10.1097/MD.0000000000025237, PMID: 33847621 PMC8052035

[3] Akamatsu S Inoue T Ogawa O Gleave ME. Clinical and molecular features of treatment-related neuroendocrine prostate cancer. Int J Urol. (2018) 25:345–51. doi: 10.1111/iju.13526, PMID: 29396873

[4] Beltran H Hruszkewycz A Scher HI Hildesheim J Isaacs J Yu EY. The role of lineage plasticity in prostate cancer therapy resistance. Clin Cancer Res. (2019) 25:6916–24. doi: 10.1158/1078-0432.CCR-19-1423, PMID: 31363002 PMC6891154

[5] Thankamony AP Subbalakshmi AR Jolly MK Nair R. Lineage plasticity in cancer: The tale of a skin-walker. Cancers (Basel). (2021) 13:3602. doi: 10.3390/cancers13143602, PMID: 34298815 PMC8306016

[6] Humphrey PA. Histological variants of prostatic carcinoma and their significance. Histopathology. (2012) 60:59–74. doi: 10.1111/j.1365-2559.2011.04039.x, PMID: 22212078

[7] Zhao SG Chen WS Li H Foye A Zhang M Sjöström M. The DNA methylation landscape of advanced prostate cancer. Nat Genet. (2020) 52:778–89. doi: 10.1038/s41588-020-0648-8, PMID: 32661416 PMC7454228

[8] Li X Chen L Peng X Zhan X. Progress of tumor-associated macrophages in the epithelial-mesenchymal transition of tumor. Front Oncol. (2022) 12:911410. doi: 10.3389/fonc.2022.911410, PMID: 35965509 PMC9366252

[9] Górska A Mazur AJ. Integrin-linked kinase (ILK): the known vs. the unknown and perspectives. Cell Mol Life Sci. (2022) 79:100. doi: 10.1007/s00018-021-04104-1, PMID: 35089438 PMC8799556

[10] Mishra AK Banday S Bharadwaj R Ali A Rashid R Kulshreshtha A. Macrophages as a potential immunotherapeutic target in solid cancers. Vaccines (Basel). (2022) 11:3390. doi: 10.3390/vaccines11010055, PMID: 36679900 PMC9863216

[11] Milbury CA Creeden J Yip WK Smith DL Pattani V Maxwell K. Clinical and analytical validation of FoundationOne^®^CDx, a comprehensive genomic profiling assay for solid tumors. PLoS One. (2022) 17:e0264138. doi: 10.1371/journal.pone.0264138, PMID: 35294956 PMC8926248

[12] Beltran H Prandi D Mosquera JM Benelli M Puca L Cyrta J. Divergent clonal evolution of castration-resistant neuroendocrine prostate cancer. Nat Med. (2016) 22:298–305. doi: 10.1038/nm.4045, PMID: 26855148 PMC4777652

[13] Imamura J Ganguly S Muskara A Liao RS Nguyen JK Weight C. Lineage plasticity and treatment resistance in prostate cancer: the intersection of genetics, epigenetics, and evolution. Front Endocrinol (Lausanne). (2023) 14:1191311. doi: 10.3389/fendo.2023.1191311, PMID: 37455903 PMC10349394

[14] Mazzucchelli R Lopez-Beltran A Cheng L Scarpelli M Kirkali Z Montironi R. Rare and unusual histological variants of prostatic carcinoma: clinical significance. BJU Int. (2008) 102:1369–74. doi: 10.1111/j.1464-410X.2008.08074.x, PMID: 18793296

[15] Rodrigues DN Hazell S Miranda S Crespo M Fisher C de Bono JS. Sarcomatoid carcinoma of the prostate: ERG fluorescence *in-situ* hybridization confirms epithelial origin. Histopathology. (2015) 66:898–901. doi: 10.1111/his.12493, PMID: 25041380

[16] Lotan TL Gupta NS Wang W Toubaji A Haffner MC Chaux A. ERG gene rearrangements are common in prostatic small cell carcinomas. Mod Pathol. (2011) 24:820–8. doi: 10.1038/modpathol.2011.7, PMID: 21336263 PMC3484363

[17] Ho GY Kyran EL Bedo J Wakefield MJ Ennis DP Mirza HB. Epithelial-to-mesenchymal transition supports ovarian carcinosarcoma tumorigenesis and confers sensitivity to microtubule targeting with eribulin. Cancer Res. (2022) 82:4457–73. doi: 10.1158/0008-5472.CAN-21-4012, PMID: 36206301 PMC9716257

[18] Tam WL Weinberg RA. The epigenetics of epithelial-mesenchymal plasticity in cancer. Nat Med. (2013) 19:1438–49. doi: 10.1038/nm.3336, PMID: 24202396 PMC4190672

[19] Nakad Borrego S Lengyel E Kurnit KC. Molecular characterizations of gynecologic carcinosarcomas: A focus on the immune microenvironment. Cancers (Basel). (2022) 14:4465. doi: 10.3390/cancers14184465, PMID: 36139624 PMC9497294

[20] Bogani G Ray-Coquard I Concin N Ngoi NYL Morice P Caruso G. Endometrial carcinosarcoma. Int J Gynecol Cancer. (2023) 33:147–74. doi: 10.1136/ijgc-2022-004073, PMID: 36585027

[21] Zając AE Czarnecka AM Rutkowski P. The role of macrophages in sarcoma tumor microenvironment and treatment. Cancers (Basel). (2023) 15:5294. doi: 10.3390/cancers15215294, PMID: 37958467 PMC10648209

